# Comment on Rodrigues, A.L.M.; Aazh, H. Psychiatric Comorbidities in Hyperacusis and Misophonia: A Systematic Review. *Audiol. Res.* 2025, *15*, 101

**DOI:** 10.3390/audiolres15060146

**Published:** 2025-10-30

**Authors:** Pawel J. Jastreboff

**Affiliations:** Department of Otolaryngology, Emory University School of Medicine, Atlanta, GA 30308, USA; jhdf2008@gmail.com

I am writing with concerns about an interesting recent publication by Rodrigues and Aazh [1] in *Audiology Research*. A crucial clarification is needed regarding the classification of depression and anxiety used in the article, specifically the differentiation between situation-evoked and chronic conditions.

Both the ICD-10-CM (the International Classification of Diseases, Tenth Revision, Clinical Modification, used to code and classify medical diagnoses) and the updated ICD-11 system distinguish between these two types of conditions. **Situational-Evoked (Reactive) depression** is categorized as an “Adjustment Disorder” (ICD-10 codes F43.21, F43.23; ICD-11 codes 6B41, 6B43.Z), a temporary reaction to a specific stressor. In contrast, **chronic depression** is classified as “Major Depressive Disorder” (ICD-10 codes F32.0–F32.3; ICD-11 codes 6A70.3, 6A71.3), which is a more pervasive, long-term condition. Similarly, **Generalized Anxiety Disorder** (GAD) (ICD-10 code F41.1; ICD-11 code 6B00) is reserved for persistent, non-situational anxiety [2,3,4].

Unfortunately, the commonly used standard questionnaires, the PHQ-9 and GAD-7, fail to differentiate between situation-evoked versus chronic depression and anxiety. This methodological oversight leads to the presence of depression or anxiety being used as proof that a patient has a “mental disorder”, without considering the root cause. Under this flawed reasoning, we would also have to incorrectly categorize all illnesses that evoke depression or anxiety (e.g., cancer) as mental disorders.

While psychologists and psychiatrists recognize the difference between situation-induced and physiologically based depression and anxiety, there is a troubling lack of evidence that this distinction is consistently applied in misophonia research, literature, or treatment. Because these two types of emotional problems have different underlying mechanisms and demand different therapeutic approaches, distinguishing between them clearly is essential for both clinical success and meaningful research.

In our own clinical experience, nearly all patients with significant misophonia also have depression and anxiety. However, in-depth interviews performed by M.D.s show that, for the vast majority, these issues developed after the onset of misophonia and are a direct result of misophonia.

Based on comprehensive medical history interviews conducted by an M.D. for all patients treated at the University of Maryland in Baltimore and later at Emory University, it was found that the vast majority of patients had Situational-Evoked depression and anxiety. But out of 318 misophonia patients, only 7 (2.2%) were diagnosed with a psychiatric disorder [5].

Making a clear distinction between situation-evoked and chronic depression and anxiety is crucial for both clinical treatment and research. It is important that results are presented with this key difference in mind. The lack of differentiation in the Rodrigues and Aazh article may lead to an overestimation of the prevalence of depression and anxiety in misophonia patients. We would appreciate it if the authors would comment on this point and clarify whether this distinction was considered.

Furthermore, the wide range of the prevalence of reported comorbidities suggests potential biases in the selection of the subject population and in the methodologies used, making it difficult to interpret the results. I would also welcome the authors’ comments on the limitations imposed by these issues.
